# 

**Published:** 1899-09-16

**Authors:** 


					TEE HOSPITAL. ?? The Standard of highest purity."? Lancet. September 10th, 1899.
CADBURY'8 TU IP* CADBURY'S
IV MM JACOCOA
COOOA
assolut*lt pur*.
COCOA
NO DRUGS OR ALKAL1E8 USED
nwrnu u am bit**.
%Vfr^SS*^fpLAscotv Western Infirmary
No. 677VVol. XXYI. [^^?]
Saturday, Sept. 16th, 1899.
[BuhsonptionTenns,] pBICE TWOPENCE.

				

